# Osteoinductive chemically crosslinked hydrogel enables hydroxyapatite formation, enhanced by release of dexamethasone, strontium, and zinc, and exhibits antimicrobial properties

**DOI:** 10.1039/d5ra09258b

**Published:** 2026-04-08

**Authors:** Benjamin Gambrill, Lirong Yang, Polina Prokopovich

**Affiliations:** a Cardiff University School of Pharmacy and Pharmaceutical Sciences Redwood Building, King Edward VII Ave Cardiff CF10 3NB UK prokopovichp@cf.ac.uk

## Abstract

Injectable hydrogels have the ability to bridge sites of bone fractures, favouring bone regeneration processes such as osteoinduction followed by osteoconduction, thereby restoring bone functions in terms of structural stability and support. The ideal material would also prevent the development of infections. Osteoinductive, osteoconductive, and antimicrobial hydrogels have not been developed yet. A hydrogel was prepared using poly(ethylene glycol) methyl ether methacrylate and 2-(dimethylamino)ethyl methacrylate, with bis[2-(methacryloyloxy)ethyl] phosphate as a crosslinker; dexamethasone and Sr and Zn ions were also incorporated. Dexamethasone and Sr and Zn ions were added into the hydrogel to encourage both bone regeneration and provide an antibacterial component, given the osteogenic/antimicrobial properties of these chemicals. The hydrogels were formulated using a crosslinker to create a rigid hydrogel capable of supporting bone regeneration while releasing the incorporated chemicals. Hydrogel polymerisation was characterised by pH and temperature changes. Hydrogel elasticity, stiffness, and viscosity were tested using a frequency sweep. The release of Sr and Zn ions and dexamethasone from hydrogels with different levels of crosslinker was also measured. The presence of hydroxyapatite, its deposition by osteoblasts and their growth, and the presence of osteocalcin were determined. The antibacterial effects were tested using *Staphylococcus epidermidis* and methicillin-resistant *Staphylococcus aureus* (MRSA). The results indicate that hydrogel swelling and changes in pH, temperature, and storage/loss modulus were favourable for the functional hydrogel, which could be injected at the fracture site. Osteoblast growth and hydroxyapatite formation were favoured by lower crosslinker concentrations, possibly influenced by the diffusion of Sr and Zn ions and dexamethasone through the hydrogel, while general cell viability was favoured by higher crosslinker concentrations. A slight antibacterial effect was observed for Sr- and Zn-releasing hydrogels compared to the standard hydrogel. The prepared hydrogel shows suitability as a material to bridge fracture sites while supporting osteoinduction in the presence of hydroxyapatite and osteocalcin and has the ability to prevent fracture-associated infections.

## Abstract 2

Injectable hydrogels have the ability to bridge sites of bone fractures, favouring bone regeneration processes such as osteoinduction followed by osteoconduction, thereby restoring bone functions in terms of structural stability and support.

## Graphical abstract

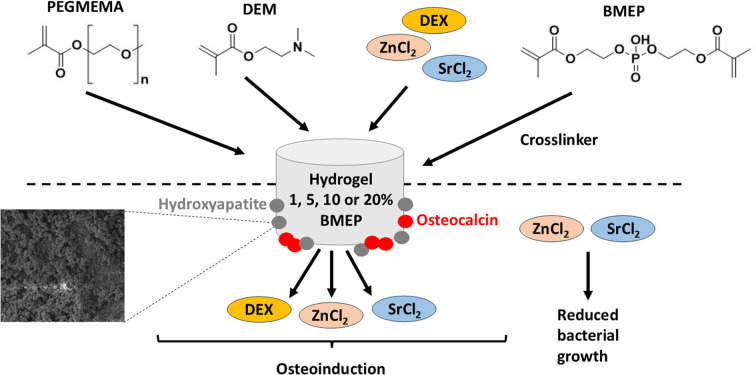

## 1 Introduction

In order to repair damaged bone, osteoinduction and osteoconduction are required processes to support mesenchymal stem cell (MSC) differentiation into osteoblasts on a surface.^1^ This is important given bone's role in providing both mechanical support for movement and protection to internal organs, particularly the skull and ribcage.^2,3^ Thus, it is crucial that when broken, bones can be quickly restored to continue performing their essential functions. There are a variety of disorders that increase the risk of bone fractures. Osteoporosis causes bone weakening with reduced bone mass and, therefore, a more disordered bone structure with increased risk of fracture.^4^ Osteogenesis imperfecta (OI) also results in compromised bones, caused by mutations in genes *COL1A1* or *COL1A2*, resulting in insufficient quantities of collagen protein production. OI patients have weaker bones, lacking the bone strength, flexibility, and support for bone mineralisation that normal amounts of collagen provide. Diseases like osteoporosis and OI, coupled with bone ageing and traumatic bone fractures through accidents, show that fractures can affect anyone, and thus, bone regeneration is an important field of research.

After treatment at fracture sites, bacterial infection may arise, where fracture-associated infections (FAIs) result in inflammatory and antibacterial responses at the wound site, and these responses are prioritised over bone regeneration, thus delaying healing.^5^ The most commonly found pathogen at fracture sites is *Staphylococcus aureus*, followed by *Staphylococcus epidermidis* and methicillin-resistant *Staphylococcus aureus* (MRSA).^6^ Therefore, another consideration for bone graft alternatives is the ability to prevent or treat infections at the fracture site. For this purpose, bone graft substitute materials containing tobramycin and silver ions encapsulated in polymethylmethacrylate^7^ and calcium phosphate beads^8^ have been tested.

For successful bone repair, bone morphogenic proteins (BMPs) produced by osteoblasts are essential, as they enable osteoinduction and subsequent bone repair and are added to bone regenerating materials. Healthy bone contains both collagen fibrils and hydroxyapatite (HA) bone crystals,^9^ which are formed of calcium and phosphate and released from osteoblasts.^10^

Given the importance of HAp, several attempts have been made to develop materials containing HAp, including bone cements with Sr-doped HAp nanoparticles, which have shown to improve cell adhesion, compressive strength and biocompatibility.^11,12^ In the design of materials suitable for bone grafts, there is great interest in polymer-based materials, which have the advantages of being biodegradable, biocompatible and non-toxic.^13^ Hydrogels, although not inherently osteogenic, have several benefits that make them suitable as bone tissue engineering substrates: materials pliable enough to bridge sites of bone fractures or defects;^14^ stiffness suitable for supporting osteoblasts and fibroblasts for osseointegration;^15^ porosity to support cell–cell signalling^16^ and surface roughness to increase surface area for bone cell attachment.^17^ Given these factors, hydrogels have been modified to enhance their potential as bone tissue engineering substrates.^18^ Minerals such as strontium and Zn have been added to hydrogels to improve bone generation support, while the inclusion of silver has shown antimicrobial properties.^19^ However, hydrogels concurrently supporting MCS differentiation into osteoblasts and HA formation, preventing microbial growth and stimulating osteoblast growth have not yet been achieved.

The aim of this study was to create a polymer-based hydrogel material with osteoinductive properties, suitable for injection at sites requiring bone regeneration, and with antimicrobial properties. The hydrogel was formulated using a bis[2-(methacryloyloxy)ethyl] phosphate (BMEP) crosslinker to produce porous hydrogels, containing Sr and Zn ions, and dexamethasone (DEX) to encourage osteoinduction, followed by osteoconduction. The inclusion of strontium (Sr) and zinc (Zn) provides the material with both antimicrobial and bone regeneration processes.^20^ Sr promotes osteoblast proliferation and inhibits osteoclast function,^21^ while Zn inhibits osteoclast activity by inhibiting osteoclast cell formation and promoting osteoclast apoptosis.^22^ DEX, usually used for rheumatoid arthritis, has been found to be osteoinductive, inducing differentiation of MSCs.^23^ Four concentrations of crosslinker (CL) BMEP were trialled to assess how material stiffness affects osteoinduction in the presence of HAp and osteoblasts. It was hypothesised that material stiffness resulting from the CL affects the release of Sr, Zn and DEX and hence the outcomes associated with their release. It was crucial that the prepared hydrogel material was non-cytotoxic to avoid bone regeneration processes while possessing antimicrobial properties to reduce the likelihood of FAIs. Thus, the hydrogel formulation presented herein provides a new potential intervention in the repair of bone fractures, with this preparation providing a means of encouraging bone regeneration while limiting the incidence of infection.

## 2 Experimental

### 2.1 Materials

PEGMEM [poly(ethylene glycol) methyl ether methacrylate] with an average Mn = 300, DEM [2-(dimethylamino)ethyl methacrylate], bis[2-(methacryloyloxy)ethyl]phosphate (BMEP), initiator ammonium persulfate (APS), ZnCl_2_, SrCl_2_, dexamethasone (DEX), phosphate-buffered saline (PBS), thiazolyl blue tetrazolium bromide (MTT), alizarin red, and dimethyl sulfoxide (DMSO) were purchased from Sigma-Aldrich, USA. Chemicals used to prepare simulated body fluid (SBF) and cell culture medium bottles were purchased from Fisher Scientific, USA. Brain heart infusion agar and broth powders were purchased from Oxoid, UK.

### 2.2 Hydrogel synthesis

A hydrogel was produced by mixing 2 mL PEGMEMA, 1 mL DEM, 5 mL distilled water and finally 10 mg of APS for hydrogel gelation. To create hydrogels with varying levels of crosslinking, a BMEP crosslinker (CL) at concentrations of 1%, 5%, 10% or 20% v/v was added to the hydrogel mixture. Zinc chloride (1 mM) and strontium chloride (100 mM), or both (1 mM ZnCl_2_ and 100 mM SrCl_2_), were included in the 5 mL of water used in the hydrogel mixture. Hydrogels included DEX either as part of the water component or as an oil emulsion. Water-based DEX was included by adding 1.1 mg DEX phosphate to the 5 mL water component of the hydrogel mixture. A DEX oil emulsion was created by adding 10 mg DEX to 1 mL oleic acid, after which 100 µL was added to the 5 mL water component of the hydrogel mixture, and it was well mixed prior to adding it to the other hydrogel components. Given the various hydrogel compositions, the final hydrogel mixture volume prior to gelling was approximately 8 mL.

The hydrogel mixture was poured into a modified syringe, which was held upright for 24 hours at 24 °C. After setting, the hydrogel cylinders were pushed from the syringe and cut into discs with a thickness of 10 mm. Alternatively, hydrogels were produced in well plates using a volume of the hydrogel mixture pipetted into the wells and allowed to gel for over 24 hours.

### 2.3 Temperature and pH change measurements during polymerisation

Temperature and pH changes were measured for the polymerisation of the hydrogels described in Subsection 2.2. Temperature changes were measured using a thermocouple (Heidolph EKT 3001) with measurements taken every minute for 40 minutes. Changes in pH were measured using a pH probe at minutes 1, 2 and 5, with 5 minutes being the time of solidification.

### 2.4 Strontium and zinc ion release

To test ion release, the hydrogels described in Subsection 2.2 were placed in 50 mL tubes with 6 mL PBS and the tubes were incubated at 37 °C for 21 days. Every 7 days, 1 mL of the PBS was collected and then mixed with 4 mL of distilled water. The 5 mL release media was analysed using an Optima 2100 DV optical emission spectrometer (PerkinElmer) and 28 Element Standard Primer-MS (Fisher Scientific, ME/1001/08). Strontium and zinc ions were detected using 407.8 and 206.2 nm filters, respectively.

### 2.5 DEX release

DEX release was determined using hydrogels (prepared without Sr or Zn) formed in 24 wells using 1 mL of hydrogel mixture for each well. To collect DEX released from the hydrogels, 2 mL PBS was added to each hydrogel, and at time points (24, 48, 72 hours, and 5 days), the 2 mL PBS was removed and replaced with fresh PBS. The quantities of released DEX were determined using HPLC (Agilent 1100 series).

### 2.6 Hydrogel swelling

To test hydrogel swelling, hydrogel discs prepared using all crosslinker concentrations without Sr or Zn were placed in 20 mL tubes with 5 mL PBS, and the tubes were incubated at 37 °C for 30 days. At each selected interval, the discs were dab-dried to remove the surface PBS and weighed; the hydrogel weight was compared with the weight of the hydrogel produced, rather than to the dried hydrogel.

### 2.7 Hydroxyapatite formation

To test hydroxyapatite formation, hydrogel discs prepared using all crosslinker concentrations, without Sr or Zn, were placed in 50 mL tubes with 10 mL SBF. SBF was described previously and comprised 8.0 mg per mL NaCl, 0.4 mg per mL NaHCO_3_, 0.2 mg per mL KCl, 0.2 mg per mL K_2_HPO_4_·3H_2_O, 0.3 mg per mL MgCl_2_·6H_2_O, 40 µL 1 M HCl, 0.3 mg per mL CaCl_2_, 0.1 Na_2_SO_4_, and 6.1 mg per mL TRIS. SBF was buffered to pH 7.4, after which the discs were incubated in 10 mL SBF at 37 °C for up to 3 months. Discs were removed from SBF after 1, 2 and 3 months and freeze dried at −40 °C for 24 hours using an Edwards Modulyo Freeze Dryer. Hydroxyapatite formation on the discs was quantified using a Philips PW1710 Automated Powder Diffractometer, and samples were run over the 2*θ* range of 10–60 at a scan speed of 0.016° 2*θ* per second. To visualise hydroxyapatite formation, SEM (Gemini, Oxford Instruments, Zeiss 1540xb) was used on discs following 2 months of incubation with SBF, compared to non-incubated samples.

### 2.8 Rheology

On a 20 mm aluminium parallel plate, 100 µL of the hydrogel mixture was used to obtain a 3 mm gap. Hydrogels were tested on an AR-G2 double head rheometer (TA instruments) with the temperature maintained at 37 °C using a frequency of 1 Hz and a strain of 1%. The storage and loss modulus were measured initially through the polymerization reaction at 6-second intervals for around 20 min until the storage modulus levelled off, indicating complete polymerisation. Immediately following complete polymerisation, the storage and loss modulus were measured through a frequency sweep at 0.01, 0.1, 1.0 and 10 Hz, with 10 increments between each frequency, and a 1% strain at 24 °C.

Hydrogel discs were suspended in 5 mL SBF and stored at 37 °C for 2 months, after which the discs were cut into different-sized discs of 25 mm diameter and 3 mm thickness. These discs were tested using an ARES-G2-II rheometer (TA instruments) with a 25 mm aluminium parallel plate. The storage and loss modulus were measured through a frequency sweep at 0.01, 0.1, 1.0 and 10 Hz, with 10 increments between frequencies at 24 °C.

### 2.9 Cell culture

The MC3T3-E1 osteoblast precursor cell line (ATCC® CRL-2593™) was selected as a standard model to assess osteogenesis from C57BL/6 mouse calvaria, resulting in the formation of mature osteoblasts and osteocytes.^24^ Rat bone marrow mesenchymal stem cells (rBM-MSCs) were selected as cells that can form mineralized bone tissues, which are important in repairing bone fractures by differentiating into osteoblasts.^25^ rBM-MSCs were obtained from 28-day-old male Wistar rats (Charles River UK Ltd, Kent, UK), as previously described.^26^ Osteoblasts (MC3T3-E1) were cultured in Dulbecco's Modified Eagle Medium (DMEM) containing 10% v/v foetal bovine serum (FBS) and 1% v/v penicillin/streptomycin. rBM-MSCs were cultured in αMEM (1X Alpha minimum essential media) containing 20% v/v FBS, 1% v/v penicillin/streptomycin, and 1% 2-phospho-l-ascorbic acid trisodium salt. Both cell lines were maintained at 37 °C in a 90% humidified atmosphere of 5% CO_2_, and the medium was removed from the cells and replaced with fresh medium every 2–3 days. Monolayers of confluent MC3T3-E1 and rBM-MSCs in T25 flasks were sub-cultured by treatment with 1 mL trypsin and 1 mL accutase, respectively, and incubated at 37 °C for 5 min. Slight agitation was applied to the cell suspensions by gently tapping the culture flask to detach the cells, which were observed to detach under the microscope. Protease activity was inhibited in the MC3T3-E1 and rBM-MSC lines by the addition of 1 mL of DMEM and 2 mL of αMEM, respectively. Each cell line was seeded into fresh T75 flasks and resuspended in a total volume of 5 mL DMEM for MC3T3-E1 and 5 mL αMEM for rBM-MSCs. For the subsequent assays, a hydrogel was used in 96 well plates using 100 µL hydrogel formulation, which was incubated at 24 °C for 24 hours.

#### 2.9.1. MTT assay

Osteoblasts at a density of 10 000 cells per well in 100 µl of medium were seeded on the hydrogels; after 24, 48, 72 hours, 7, 14, and 21 days, 80 µL of fresh medium was exchanged with any remaining media or added if no media was remaining. MTT assay was carried out on wells comprising hydrogels of all crosslinker and ion varieties. The MTT stock solution was prepared by mixing 5 mg per mL thiazolyl blue tetrazolium bromide in PBS, of which 10 µL was added to each well, and the plates were incubated at 37 °C for 2 hours. Following incubation, 50 µL of DMSO was pipetted into each well and retained for 10 min. For analysis, 80 µL from each well was taken and transferred into a fresh 96-well plate. The absorbance was measured three times at 570 nm using a Labtech-LT5000MS ELISA spectrophotometer according to Sigma Aldrich, UK MTT protocol.

#### 2.9.2. Alizarin red assay

Osteoblasts at a density of 10 000 cells per well in 100 µL of medium were seeded on the hydrogels; after 24 hours, 48 hours, 72 hours, 7 days, 14 days and 21 days, the media was removed and replaced with 100 µL of 10% glutaraldehyde in PBS, which was added to each well and incubated at 37 °C for 10 minutes. The glutaraldehyde was removed, and the hydrogels were washed three times using 100 µL PBS, after which 100 µL of 1% alizarin red stain (ARS) was added and retained for 20 minutes. ARS was removed, hydrogels were washed with distilled water, and then 100 µL of 10% acetic acid was added to each well and retained for 30 min. For analysis, 50 µL from each well was taken and transferred into a fresh 96-well plate. The absorbance was measured at 405 nm using a Labtech-LT5000MS ELISA reader.

#### 2.9.3. Osteocalcin protein ELISA

rBM-MSCs were dispersed in media, and 100 µL was added to each well for a final density of 10 000 cells per well. Every 3 days, 80 µL of fresh medium was exchanged with any remaining media or added if no media remained. After 21 days, the media was collected, and 25 µL was used with the osteocalcin ELISA kit (Invitrogen) by pipetting the media into the OST antibody-coated microtiter strip well plate. For the reaction, 100 µL of Anti-OST-HRP conjugate was added to each well and incubated at 24 °C for 2 hours; then, 100 µL of stabilized chromagen was added and further incubated at 24 °C for 30 min. To terminate the reaction, 100 µL of stop solution was added to each well, and the absorbance was read at 450 nm with a Labtech-LT5000MS ELISA reader. The osteocalcin concentration was determined using a standard curve generated using the standards provided in the kit.

### 2.10 Microbiology

In 24 well plates, 1 mL of hydrogel compositions was used; a hydrogel with 1% crosslinker and variations in ion salts was used (100 mM Sr, 1 mM Zn, and 100 mM Sr and 1 mM Zn), compared with a hydrogel prepared without ions as a control. Each hydrogel formulation was used in triplicate against each of the bacteria used: methicillin-resistant *Staphylococcus aureus* (MRSA) (NCTC 12493) and *Staphylococcus epidermidis* (RP62a). Bacterial cultures were prepared by taking a single bacterial colony, inoculating 10 mL BHI broth and incubating at 37 °C for 24 hours. Moreover, 1.5 mL of the bacterial culture was added to each hydrogel, which was incubated at 37 °C for 1 hour, after which the culture was removed and the hydrogel was washed three times with 1 mL sterile PBS. To determine if bacterial growth occurs after the removal of culture, the hydrogels were incubated with 1.5 mL of a mixture comprising 10% sterile broth and 90% sterile PBS. Every 15 minutes over 24 hours, 50 µL of the mixture was collected, mixed with 100 µL sterile broth and tested for absorbance using a Bioscreen plate reader, compared with 45 µL sterile PBS and 105 µL sterile broth, to represent an appropriate control.

### 2.11 Statistical analysis

Error bars on figures represented standard deviation, where appropriate one-way ANOVA, Kruskal–Wallis or Fisher's LSD test was performed to test for significant difference between values. A *P* value of <0.05 was regarded as statistically significant.

## 3 Results and discussion

### 3.1 Hydrogel characterisation

#### 3.1.1. Change in pH during polymerisation

**Fig. 1:**
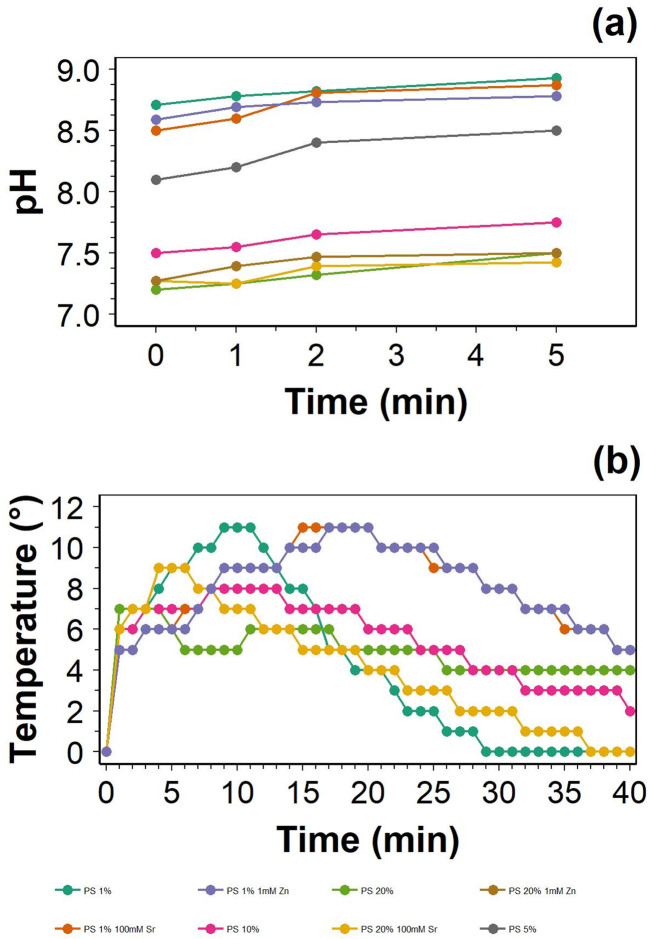
Changes in pH (a) and temperature (b) during the polymerisation process for the developed hydrogels.

Prior to hydrogel solidification, the pH measurement initially ranged from 8.7 to 7.6, with decreasing values and an increasing fraction of CL; minimal impact was observed when Sr or Zn was added (Fig. 1a). For 5 minutes prior to hydrogel solidification, the final pH values were measured as ∼8.8 for 1% CL hydrogel, and pH ∼7.5 for 20% CL hydrogel, suggesting that a greater concentration of CL results in an acidified hydrogel mixture.

The use of pH 7.4 for *in vivo* testing is appropriate because it is thought that fracture site pH is initially 7.4 (physiological pH), after which inflammation creates a more acidic environment, as discovered in bone repair models.^27^ After a few days of inflammation, the fracture site pH increases, enabling optimal conditions for osteoblasts.^28^ Taken together, this suggests that an injectable hydrogel may have difficulty producing HAp, as inflammation could prevent HAp formation until the pH returns to more alkaline conditions, which may be further delayed if FAI is present. The greatest pH value measured prior to hydrogel gelation was below pH 8.8, which represents a more alkaline environment than the fracture site pH. The pH range 8–8.4 represents the maximal pH for osteoblast-like cell proliferation^19^ and bone mineralisation,^20^ with repair processes favouring alkaline conditions, suggesting that the pH at the point of gelation is not too extreme to completely inhibit osteoblast activity.

#### 3.1.2. Change in temperature during polymerisation

The polymerisation reaction to produce the hydrogel was most exothermic for the hydrogel with 1% crosslinker, both with and without 100 mM SrCl_2_, with an increase in temperature of up to 11 °C and a delayed reaction compared to the 100 mM 1% CL hydrogel (Fig. 1b). With an increasing fraction of CL, the temperature increase reached a maximum earlier but with a smaller temperature increase. The highest temperature increase for the 20% CL hydrogel was up to 9 °C higher than room temperature. After 40 minutes, the final recorded temperatures were 5, 0 and 4 °C above room temperature for the 100 mM SrCl_2_ 1% CL, 1% CL and 20% CL hydrogels, respectively. Thus, it may take longer than 40 minutes for 100 mM SrCl_2_ 1% CL and 20% CL hydrogel to return to room temperature, which only took 29 minutes for a 1% CL hydrogel.

Here, the hydrogel was synthesised at 24 °C and left for 24 hours prior to testing and at 37 °C for rheology testing. Polymerisation appeared completed at approximately 20 minutes for all CL concentrations at 37 °C. It is expected that at higher temperatures, hydrogel gelation would be more rapid, as demonstrated by the chitosan–polyethylene oxide–glycerol phosphate hydrogel, which takes approximately 6 minutes to gel 1 mL of the hydrogel mixture at 42 °C, despite this temperature being higher than the body temperature.^29^ The use of hydrogels for bone regeneration, as employed here, is not limited to bone regeneration applications, as hydrogels have the advantage of taking any shape required, with varying degrees of crosslinking available.^30^ The use of BMEP as a crosslinker is not novel, as its use allows for material designs with increased porosity and compressive mechanical strength.^31,32^ BMEP, once activated by the initiator (ammonium persulfate), converts the methacryloyloxy groups into free radical sites, from which interactions with PEGMEM and DEM, or other CL molecules, occur to produce covalently bonded polymer chains.^33^ Phosphorus encourages the binding of calcium ions, therefore promoting bone formation.^34^ Thus, BMEP, as a hydrogel crosslinker, plays a dual role: providing a mechanically strong material and promoting calcium binding to the material.

Bone scaffolds are usually expected to be resorbable; however, the appropriate rate of scaffold resorption is still debatable, with suggestions ranging from 2–3 weeks to 3–6 months.^35^ The appropriate degradation rate is important, as a degradation rate faster than cellular infiltration can lead to union failure, while a degradation rate slower than the rate of infiltration can interfere with bone formation, increasing the risk of graft failure. Similar hydrogels have shown a degradation rate of ∼15% over 2–3 weeks.^35^ Thus, it is expected that the degradation rate *in vivo* is in a suitable range.

#### 3.1.3. DEX release from hydrogels

**Fig. 2:**
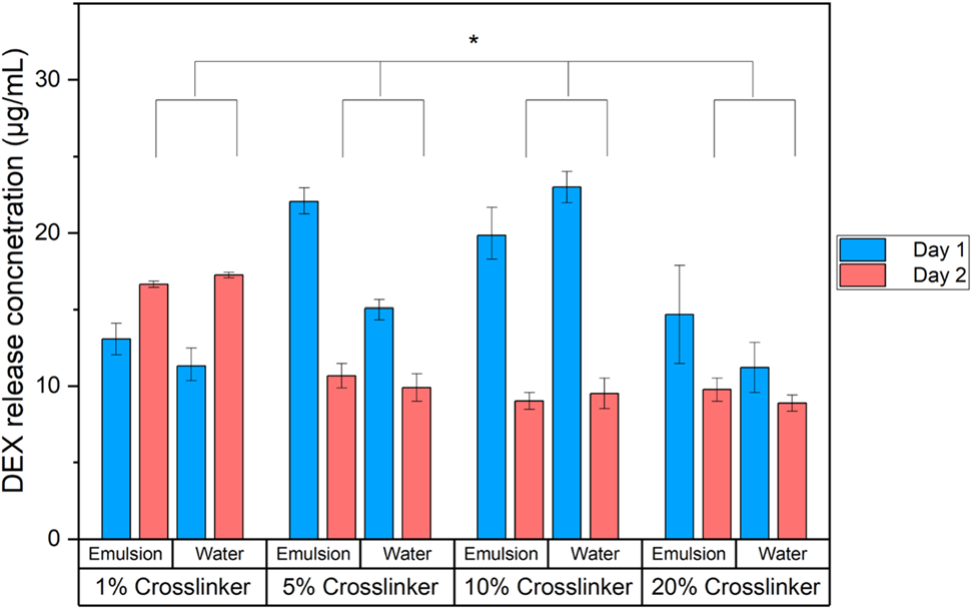
DEX release from CL hydrogels, from water or oil emulsion of DEX. Asterisk signifies *P* ≤ 0.05.

The concentration of DEX release from hydrogels, either emulsion or water encapsulation of DEX, showed no difference for either type of DEX encapsulation for each crosslinker group (two-way ANOVA, *P* = 0.02) after 1 or 2 days. The greatest release of DEX was measured from 10% crosslinked hydrogel, followed by 5% after 1 day of incubation in PBS (Fig. 2). After collecting the second release samples representing day 2 DEX release, the greatest release was measured for the 1% CL hydrogel for both DEX encapsulation methods. The release of DEX from the hydrogel is governed by mass transport principles; DEX phosphate is water soluble and is thus added directly to the hydrogels. DEX is not water soluble and is solubilised in oleic acid, allowing the formation of a water/oil emulsion. The release of DEX phosphate is regulated by its diffusion coefficient in the hydrogels, while DEX from the emulsion first has to migrate from the oil droplet to the water phase of the gel before it is released. The generally higher release observed from the hydrogels containing DEX in emulsion compared to hydrogels with DEX phosphate could be due to the destabilisation of the emulsions during the polymerisation reaction in light of the temperature increase. Preparation of hydrogels with emulsions requires two steps in the synthesis (dissolution of DEX in oleic acid and preparation of the emulsion). Thus, it would be justified only if the release from DEX phosphate was not satisfactory; it is, therefore, apparent that the use of emulsions to prepare DEX releasing hydrogels is preferable over the use of DEX phosphate.

#### 3.1.4. Sr and Zn release from hydrogels

**Fig. 3:**
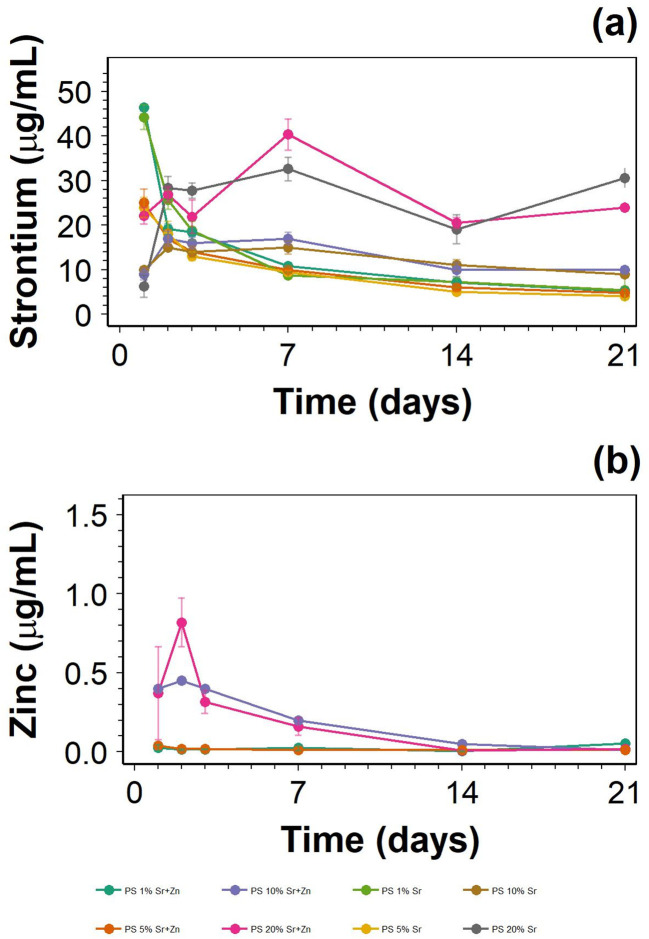
Strontium (a) and zinc (b) release from hydrogels over 3 weeks.

The greatest Sr release was measured from hydrogels containing 100 mM SrCl_2_ and, 100 mM SrCl_2_ and 1 mM ZnCl_2_. After 1 day, the amounts of SrCl_2_ released from 1% CL hydrogels containing 100 mM SrCl_2_, as well as those containing 100 mM SrCl_2_ and 1 mM ZnCl_2_, decreased each day (Fig. 3a). The release of Sr from hydrogels with 100 mM SrCl_2_ and, 100 mM SrCl_2_ and 1 mM ZnCl_2_ with increasing % CL was delayed compared to 1% CL hydrogels, suggesting that higher CL concentrations result in slower diffusion of ions through the hydrogel.

The greatest Zn release was measured on day 2 for the 20% CL hydrogel with 100 mM SrCl_2_ and 1 mM ZnCl_2_ (Fig. 3b). Zn released from 10% CL hydrogels was similar to that released from 20% CL; apart from these instances, there was very little Zn release, suggesting that Zn does not diffuse easily through the hydrogel.

Both Sr and Zn are important for normal bone function; Sr promotes osteoblast proliferation and inhibits osteoclast function,^21^ while Zn inhibits osteoclast activity by inhibiting osteoclast cell formation and promoting osteoclast apoptosis.^22^ Therefore, the inclusion of Sr and Zn is justified with the aim of promoting osteoblast function in the restoration of bone. For antibacterial hydrogels, Zn, as used here, is used in the form of nanoparticles released from a chitosan hydrogel crosslinked using glycerol phosphate.^29^ Greater release of Sr and Zn ions, and DEX from 20% CL hydrogel as opposed to hydrogel with lesser CL concentration could be explained by phase separation. Phase separation occurs when hydrophobic and hydrophilic polymer networks interact with one another, excluding water from the formed matrices.^36^ Sr and Zn ions, and DEX are water soluble and could exist in water phases; in hydrogel with lesser CL concentrations, the water-soluble Sr and Zn ions, and DEX are more evenly spread throughout the hydrogel, compared to high CL concentration hydrogels, which polymerise more rapidly, possibly creating aggregates of Sr and Zn ions, and DEX within the hydrogel. Although potentially limiting the release of Sr and Zn ions, and DEX here, phase separation has been exploited for hydrogel-based delivery systems.^37–39^ It is also possible that the highest swelling observed in the low CL hydrogels results in a decreased concentration of the ions in the hydrogels, thus reducing the mass transfer process driven by differences in concentrations between the bulk of the hydrogel and the liquid phase.

#### 3.1.5. Hydroxyapatite formation on hydrogels

**Fig. 4:**
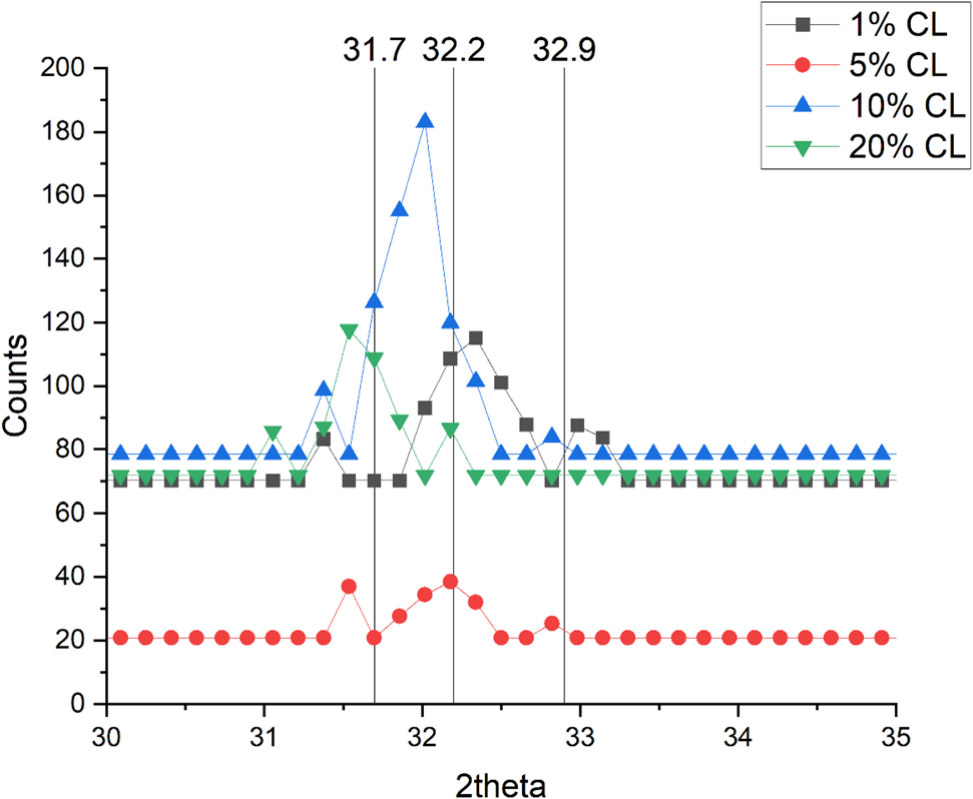
HAp formation on each hydrogel (assessed using X-ray diffraction) after 2 months, for hydrogels with different crosslinker concentrations. Vertical lines show expected peaks corresponding to presence of HAp.^40^

**Fig. 5:**
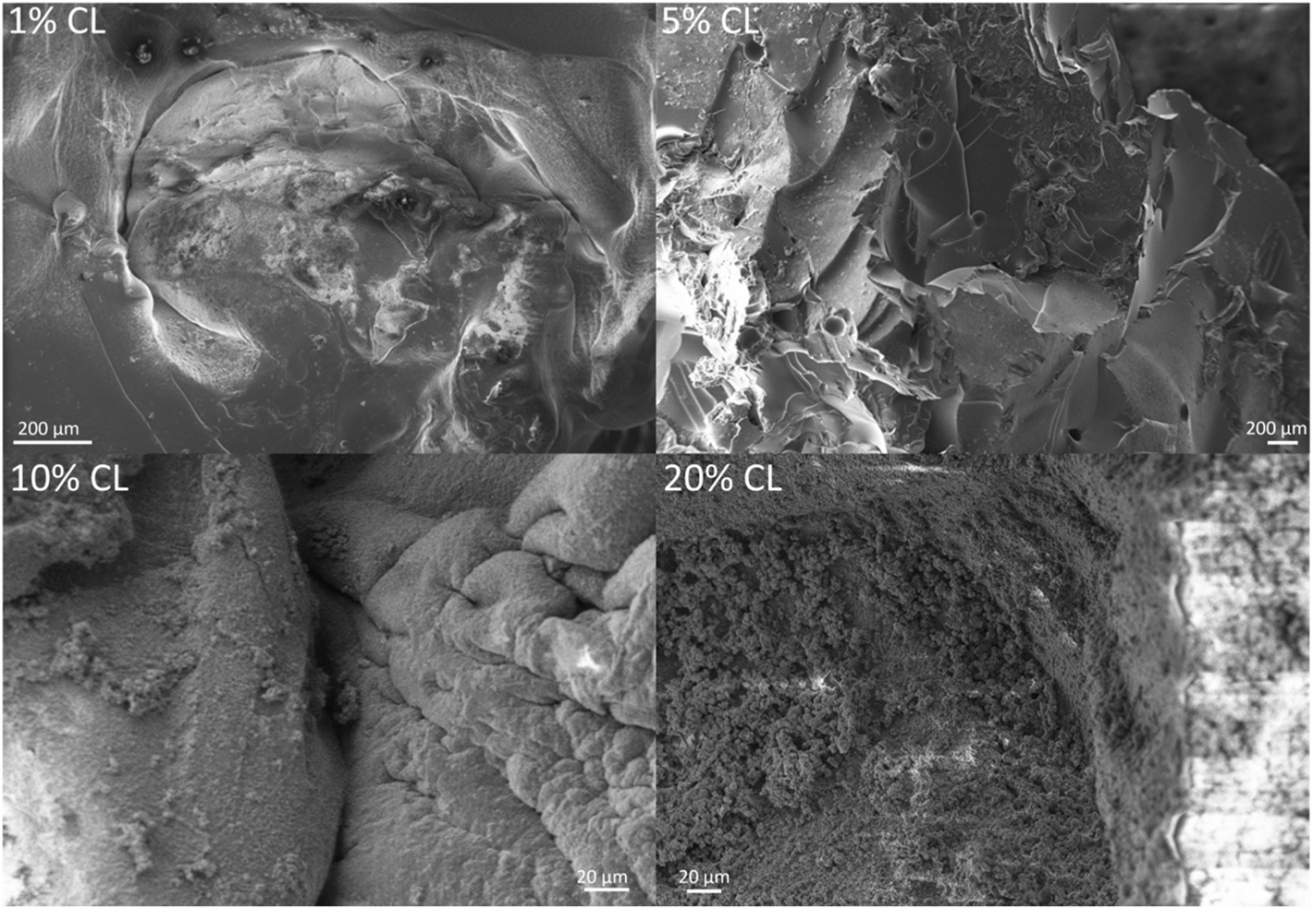
SEM images of HAp formation on the hydrogel formulated using different crosslinker concentrations.

The use of SBF to encourage HAp formation on the hydrogels demonstrated that HAp was formed on 5%, 10% and 20% CL hydrogels after 1 month, in accordance with the expected X-ray diffraction (XRD) peaks for HAp formation. XRD peaks, 2*θ* = 31.7°, 32.2° and 32.9° are expected to signify the presence of HAp formation.^40^ After 2 months, HAp was present on all concentrations of CL hydrogels, which were measured using XRD (Fig. 4) and observed using SEM images (Fig. 5). After three months of immersion in SBF, there appeared to be no HAp on the 5% CL hydrogel, with other concentrations of CL hydrogels retaining their HAp.

The use of SBF enabled the synthesis of hydroxyapatite in the hydrogel. Given that the ion concentration (Ca^2+^ and PO_4_^3−^) present in the SBF is responsible for HAp formation, increasing the ion concentration results in increased HAp formation, a process also influenced by pH.^41,42^ In this study, the pH of SBF was 7.4, which represents the favourable pH at which HAp is usually formed, with lower pH values resulting in octacalcium phosphate (OCP) formation.^43^ Both OCP and HAp are related, with OCP capable of converting to HAp in a mechanism not fully understood.^44,45^ It is postulated that OCP irreversibly hydrolyses to produce calcium-deficient HAp, which is the more thermodynamically stable of the two bone minerals.^46^ The Ca/P molar ratios of these calcium phosphates^47,48^ (OCP: 1.33, HAp: 1.67, calcium-deficient HAp: 1.55) show that the formation of HAp and calcium-deficient HAp is favourable, as calcium is vital for providing bone strength and mass.^49^ The formation of hydroxyapatite (or OCP) on the hydrogel surface could also inhibit the movement of the Sr and Zn ions used here, and therefore reduce activities associated with DEX, Sr and Zn release, as found in the study encapsulating silver nanoparticles into hydrogel formed using PEGMEM and DEM.^19^ Moreover, the use of a CL with a phosphate group improved HA deposition compared to similar hydrogels that used a different CL agent without phosphate.^50^

OCP is osteoconductive despite its weak thermostability and its conversion into HAp and is used in biomaterials, showing greater bone regeneration than HAp (or tricalcium phosphate) containing materials.^45,51,52^ To mimic bone more accurately, carbonated apatite (CO_3_Ap) should be considered alongside HAp, as carbonated apatite is more representative of the apatite found in bone,^53^ which inherently has biocompatibility and osteoconductivity.^54^ To further study the initial formation of OCP and hydrolysis into HAp upon hydrogel, attention should be focused on XRD, where the 2*θ* peak at 4.7° characteristic of OCP could be used to signify hydrolysis into Hap.^55^ The 2*θ* range employed for XRD was 10°–60°, which therefore did not allow for the assessment of OCP conversion into HAp.

#### 3.1.6. Hydrogel swelling

**Fig. 6:**
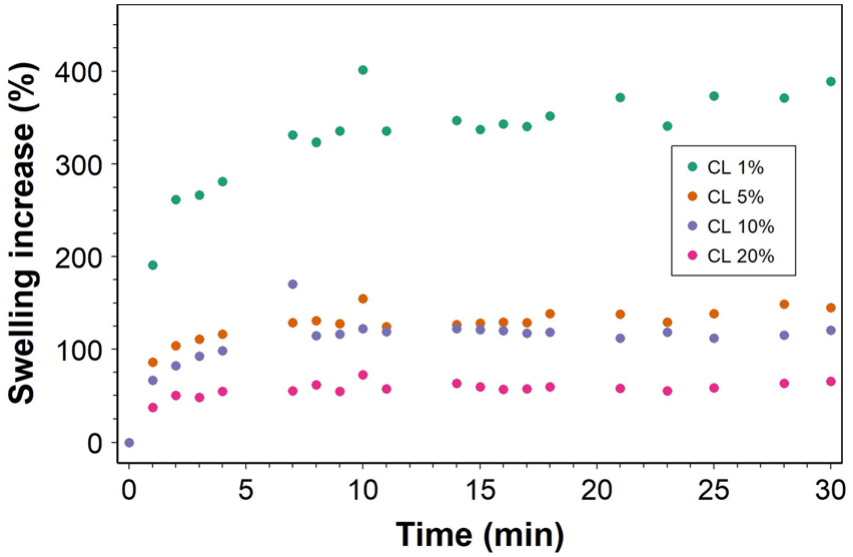
Hydrogel swelling in phosphate-buffered saline over 30 days for hydrogels with different concentrations of the crosslinker. Datapoints are the average value of three repeats for each CL concentration.

As expected, the hydrogel with the greatest swelling over 30 days was the one with the lowest concentration of CL, which was 1% (Fig. 6). Both 5% and 20% CL had similar levels of swelling, approximately doubling in weight. Surprisingly, the CL hydrogel with the least swelling was the 10% CL, where approximately only half of the original weight was added as swollen weight.

### 3.2 Hydrogel mechanical testing

**Fig. 7:**
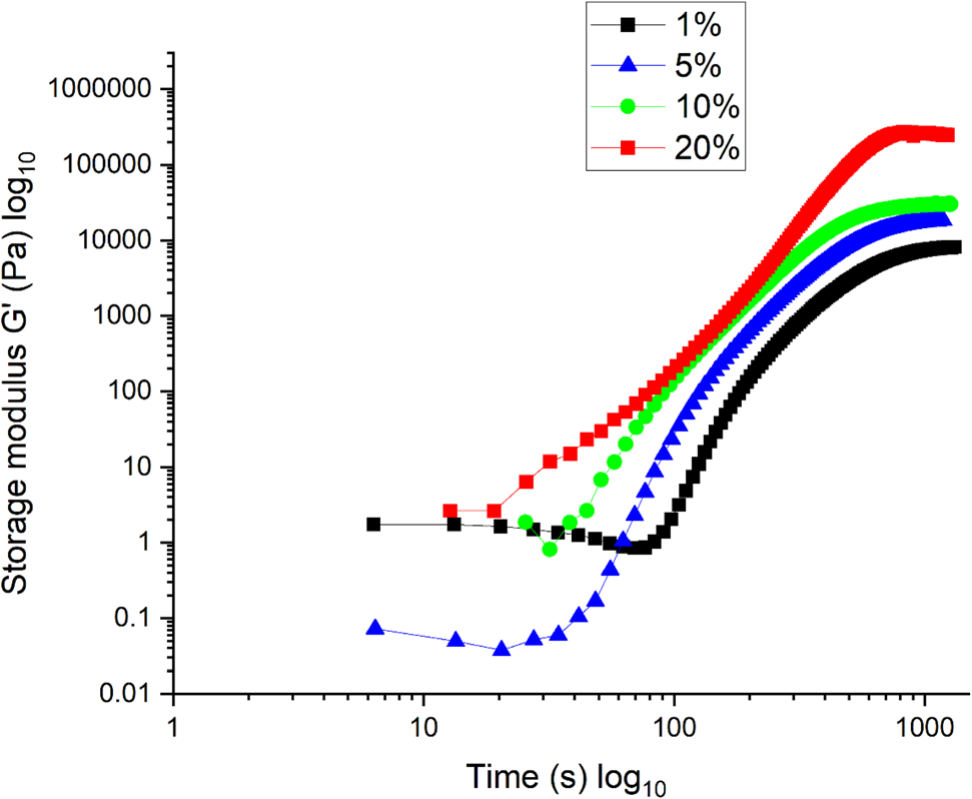
Storage modulus during hydrogel polymerisation.

It was observed that increasing the CL concentration resulted in an increased storage modulus (material stiffness) for the 20 minutes tested and a quicker reaching of the storage modulus plateau, representing the maximal stiffness possible for the hydrogel. As expected, the 1% CL hydrogel takes the longest to reach the plateau, with the least storage modulus of the four CL concentrations tested (Fig. 7). This confirms the expectation that increasing the concentration of CL increases hydrogel stiffness by increasing the number of chemical bonds made within the hydrogel.

**Fig. 8:**
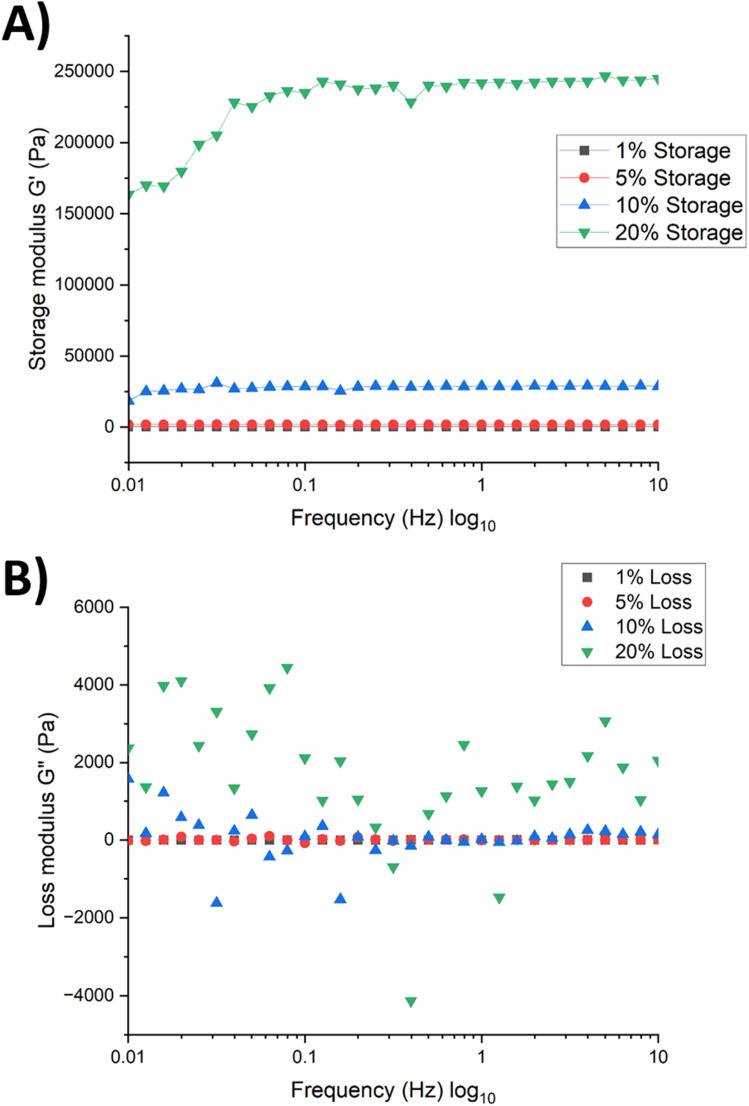
Storage (A) and loss modulus (B) tested at increasing frequency waves for different hydrogel crosslinker concentrations after polymerisation.

The storage modulus of the fully formed hydrogels was tested using a frequency sweep to test for hydrogel viscoelasticity. As with the storage modulus tested for hydrogel formation, the 20% CL had the greatest storage modulus (Fig. 8A), decreasing with CL concentration, with 10% CL having 9 times less storage modulus, and 1% and 5% CL exhibiting no Pa with a plateau across 0 Pa. The near zero Pa results indicate weak elasticity compared to the 10%, especially the 20% CL concentrate, demonstrating that increased CL concentration allows for the storage of energy and the ability of the hydrogel to maintain its shape as the frequency sweep intensity increases.

The loss modulus was found to be greatest for 20% CL hydrogel but also with the greatest variations, a trend decreasing with a CL concentration (Fig. 8B). Hydrogels with CL concentrations of 1% and 5% show a limited loss modulus, suggesting that molecular rearrangements within the hydrogel occur to a lesser extent, whereas 10% and 20% CL hydrogels exhibit fluctuating loss moduli, indicating more pronounced structural rearrangements within the hydrogel.

### 3.3 Cell culture

#### 3.3.1. MTT osteoblast cell viability assay

**Fig. 9:**
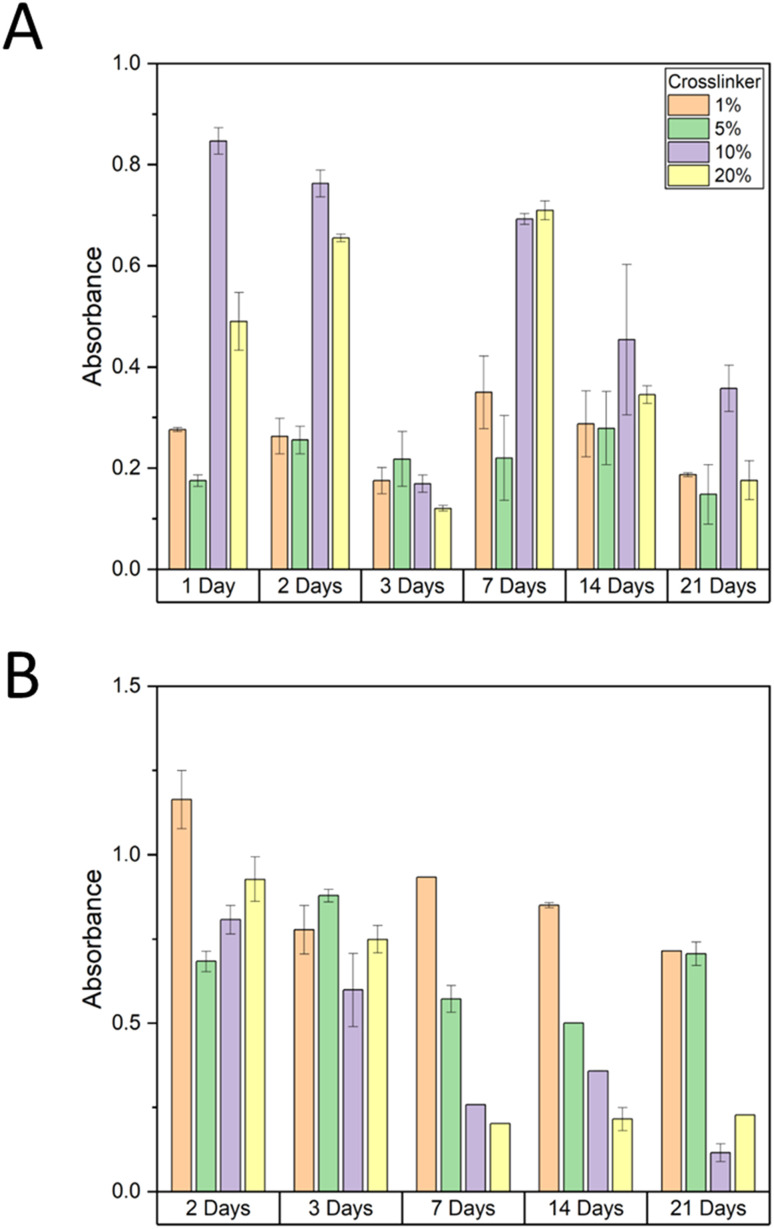
Effect of hydrogel crosslinker concentration on osteoblast (A) cell viability (MTT assay) and (B) calcium (alizarin red assay) deposition over 3 weeks. Error bars shown are standard deviation bars. *n* = 3 for each sample.

**Fig. 10:**
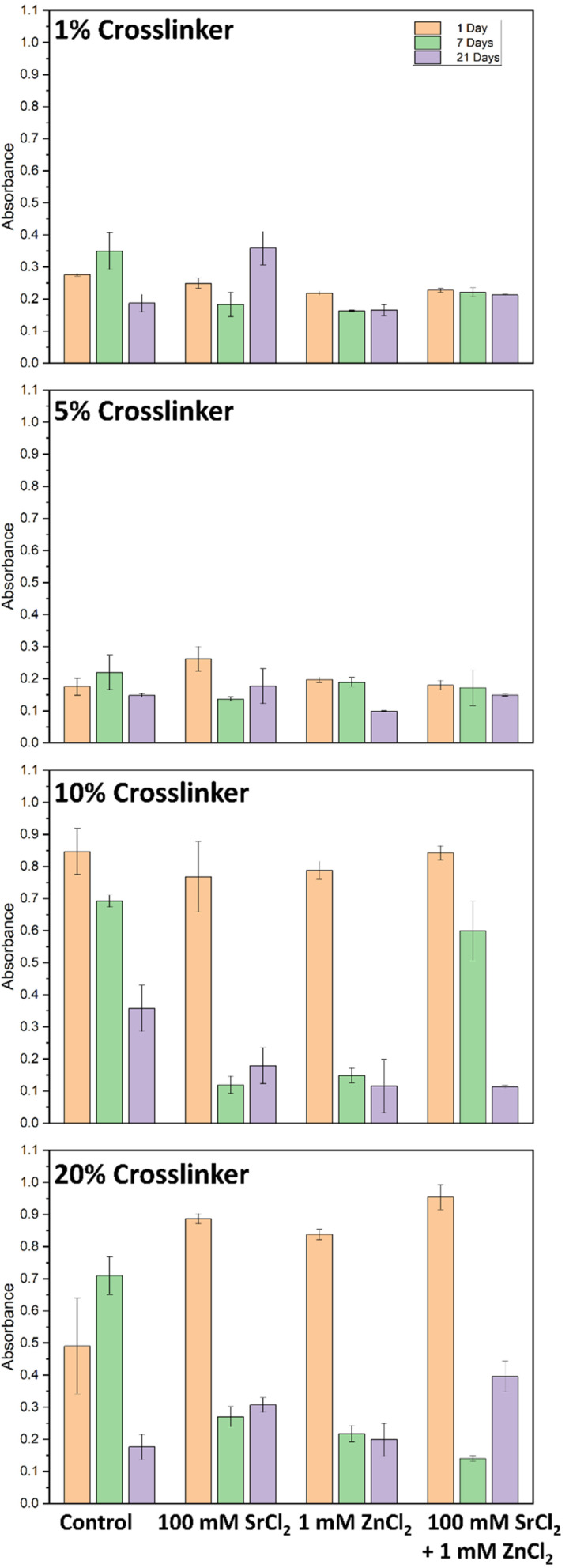
Effect of salt release from each hydrogel crosslinker concentration on osteoblast viability (MTT assay) at days 1, 7 and 21. Error bars are the standard deviation bars. *n* = 3 for each sample.

MTT assay used to measure mitochondrial activity showed increased absorbance for hydrogel with 10% and 20% crosslinkers for 1, 2, 7, 14 and 21 days (all except for 3 days) compared to hydrogel with 1% and 5% crosslinker (ANOVA and *post hoc* Tukey test, *P* = 0.005) (Fig. 9A). An MTT assay was also used to determine whether 100 mM SrCl_2_, 1 mM ZnCl_2_ or both salts at these concentrations were present in the hydrogel with each crosslinker concentration affecting cell viability or calcium deposition (ANOVA and *post hoc* Tukey test, *P* = 0.02) (Fig. 10). After 21 days there was greater cell viability for 1% crosslinker hydrogel releasing SrCl_2_ compared to no salt hydrogel with the same crosslinker concentration (Fisher's LSD test, *P* = 0.03). This could be attributed to the importance of Sr and Zn. The greatest cell viability for 5% crosslinker hydrogels was with hydrogel releasing SrCl_2_ after 24 hours. For hydrogels with 10% and 20% crosslinker, the presence of SrCl_2_ and ZnCl_2_ independently decreased cell viability for 7 and 21 days. The osteoblast viability of salt containing hydrogels was similar to hydrogels with no salt and 10% and 20% crosslinker for 7 days.

#### 3.3.2. Alizarin red calcium deposition assay

**Fig. 11:**
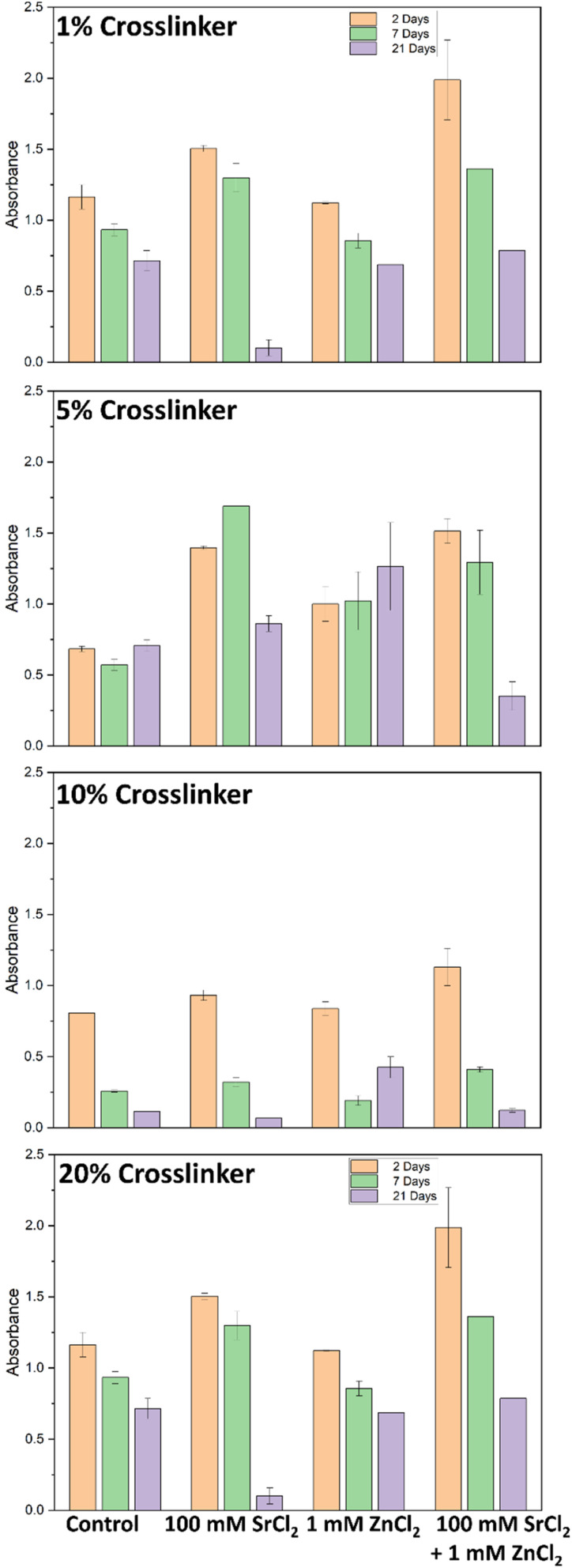
Effect of salt release from each hydrogel crosslinker concentration on osteoblast calcium (alizarin red assay) deposition at days 2, 7 and 21. Error bars are standard deviation bars. *n* = 3 for each sample.

Calcium deposition from osteoblasts was measured using the alizarin red assay, showing the greatest levels of deposition with hydrogel with 1% crosslinker, followed by 5% crosslinker. Over time, calcium deposition decreased for all hydrogel crosslinker compositions (ANOVA and *post hoc* Tukey test, *P* = 0.01) (Fig. 9B). Calcium deposition, measured using alizarin red assay, showed that the release of salts from 1% crosslinker had greater calcium deposition for all salt concentrations after 2 and 21 days, and similar levels of calcium deposited for all salts after 7 days (ANOVA and *post hoc* Tukey test, *P* = 0.01) (Fig. 11). Generally, the release of salts from 5% crosslinker hydrogels was associated with increased calcium deposition. For hydrogels with 10% and 20% CL, the presence of SrCl_2_ and ZnCl_2_ independently and together showed little or no change in calcium deposition compared with hydrogels with no salts of the same CL concentrations. It is expected that an increased CL concentration would result in less calcium deposition, as the diffusion of ions through the hydrogel would be suppressed. As discussed previously, phase separation has been exploited for hydrogel-based delivery systems,^37–39^ and in the case of surprising calcium deposition associated with higher concentrations of CL, this could be used to support the idea.

#### 3.3.3. Osteocalcin ELISA

**Fig. 12:**
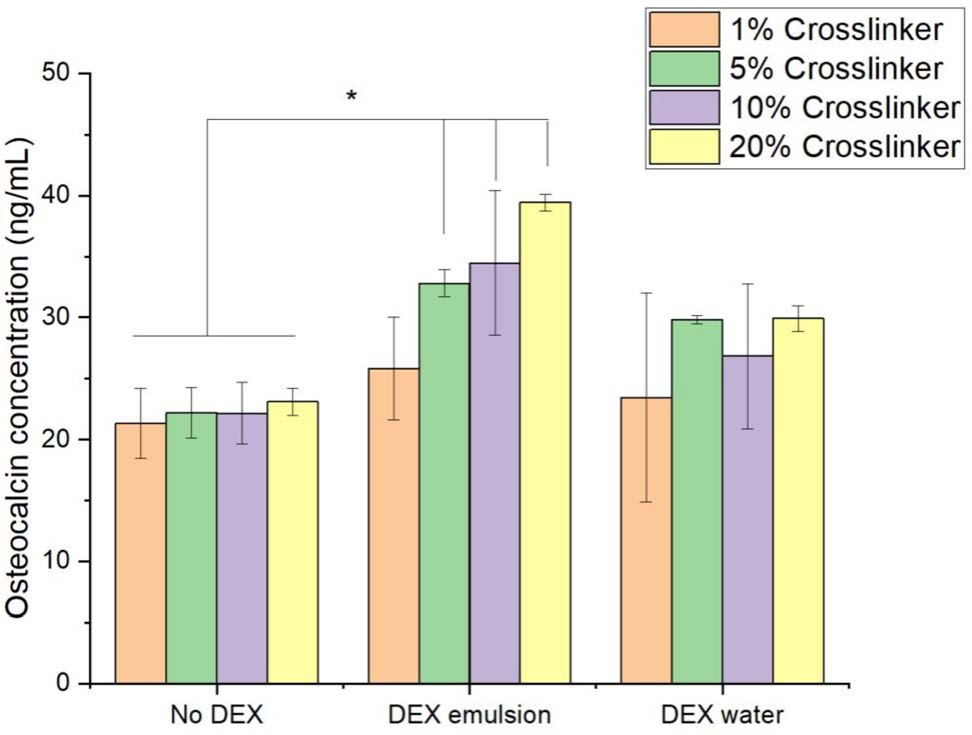
Effects of DEX and hydrogel crosslinker concentration on osteocalcin production by using rBM-MSCs. Asterisk signifies *P* ≤ 0.05. Error bars are standard deviation bars. *n* = 3 for each sample.

Similar levels of osteocalcin were found after rBM-MSC growth on any of the hydrogels without DEX (ANOVA, *P* > 0.05) (Fig. 12). When DEX was added as an oil emulsion, there was significantly more osteocalcin produced by hydrogel with 5% or more CL compared to the same hydrogel without DEX (Fisher's LSD test, *P* < 0.001), suggesting that the presence of emulsified DEX in hydrogel enables more osteocalcin to be produced. The levels of osteocalcin observed after rBM-MSC growth on any of the hydrogels containing water-based DEX were not statistically different among the different amounts of CL (ANOVA, *P* > 0.05); however, these were greater than the osteocalcin produced on the corresponding hydrogels without DEX for hydrogels with 5% or more CL (Fisher's LSD test, *P* < 0.001).

Osteoblasts were chosen as the most relevant cell line for the validation of materials supporting bone regrowth; thus, the compatibility of these cells with the developed hydrogels was assessed using the MTT assay and alizarin red assay, which are related to the osteoblast ability to produce calcium deposits, the primary components of bone. Stem cells were chosen instead to confirm the osteogenic activity of the material; thus, osteocalcin production was assessed only for MSCs, with DEX believed to increase the expression of osteocalcin by MSCs, which is involved in the leptin pathway, with DEX increasing leptin expression and decreasing osteocalcin expression.^56^ These types of cells have been routinely used in musculoskeletal biomaterial development.^57–59^

A hydrogel with an increased CL concentration was found to increase cell viability, potentially justified by a smaller surface area for successful adhesion, while an increased CL concentration decreased calcium deposition, which can be explained by a lower expected release of osteogenesis promoting ions, Sr and Zn. This provides opportunities for fine tuning CL concentrations for desirable outcomes; an ideal bone tissue engineering material should encourage osteoblasts in order to be osteoconductive without being cytotoxic. Given the low cell viability at high CL concentrations and high calcium deposition at low CL concentrations, we propose further tests using CL concentrations in the range of 5–10%, as these are the central concentrations tested.

Hydrogels with lower CL concentrations have lower densities and, therefore, greater porosity, allowing for the movement of Sr and Zn ions and DEX, which could explain the decreased calcium deposition and osteocalcin production. DEX has been widely used for bone tissue engineering applications, encouraging osteoinduction,^60^ which is favoured by a low CL concentration, as shown here. Reduced cell viability, specifically of osteoblasts,^61^ is reduced by high concentrations and diffusion of DEX, a result of low CL concentrations, also shown here. The use of DEX should be carefully considered owing to the outcomes of studies, which demonstrate that DEX usage results in osteoporosis^62^ and bone erosion.^63^

### 3.4 Microbiology

**Fig. 13:**
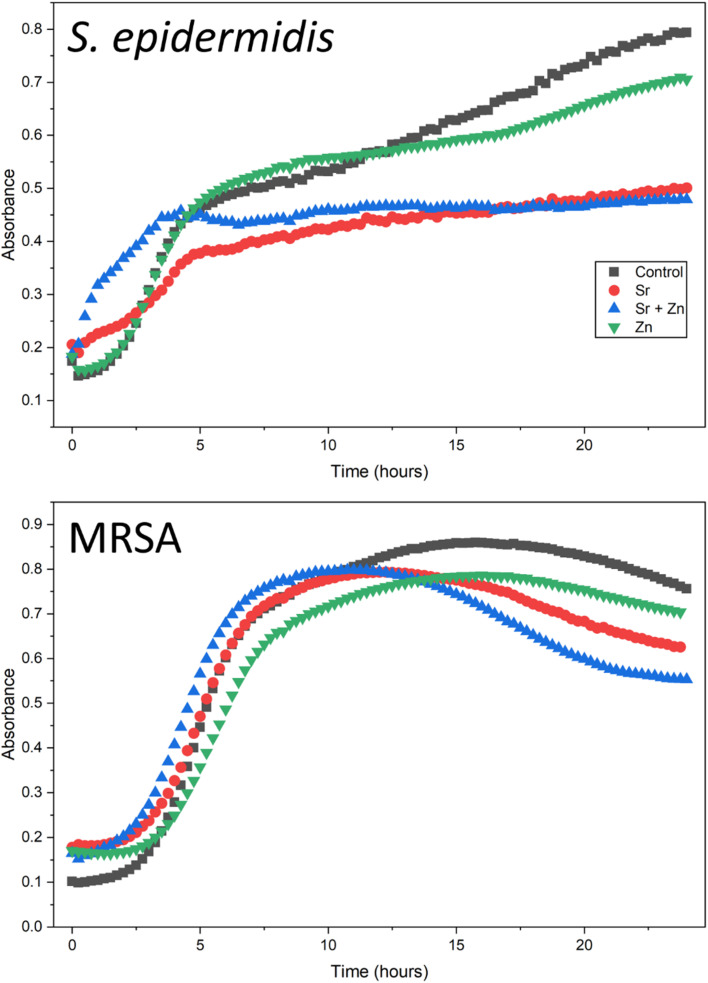
Antibacterial effect of 1%-crosslinker-concentration, salt-containing hydrogels on the *S. epidermidis* and MRSA bacterial growth.

1% CL hydrogels with Sr and Zn were chosen in light of the highest release of Sr, while Zn was released minimally. Therefore, this hydrogel provides the best osteogenic platform through Sr ions and does not impact the simultaneous release of Zn. After 24 hours, the least bacterial growth for both *Staphylococcus epidermidis* and MRSA was measured for cultures in the presence of 1% CL hydrogel with 100 mM Sr and 1 mM Zn, compared to 1% CL hydrogel (control), which had the greatest growth after 24 hours (Fig. 13). Initially, 1% CL hydrogel with Sr and Zn showed the greatest bacterial growth before 5 hours, after which the bacterial culture density plateaued. Independently, 1% CL hydrogels with Sr were more antibacterial than hydrogels with Zn for the tested bacteria. Zn and Sr ions are widely regarded for their antibacterial properties. Zn ions generate reactive oxygen species, interfere with bacterial cell walls, and affect cell morphology.^64,65^ Strontium ions can interfere with bacterial metabolic pathways;^66^ therefore, in combination with zinc ions, they have synergistic effects, as potentially indicated by the combination of Zn and Sr having the greatest antibacterial effect. However, some studies suggest that Sr does not possess antibacterial properties,^67^ and its inclusion with Zn is purely due to the antibacterial effect of Zn in combination with the osteogenic promotion offered by Sr.^68^ Either way, the presence of these ions in the hydrogel and their release support bone regeneration while eliciting an antibacterial effect.

## 4 Conclusions

The hydrogel prepared here has osteoconductive and osteoinductive properties with HAp formation and cell viability varying with the degree of crosslinking, providing opportunities for further work. The inclusion of DEX in the hydrogel somewhat improved the potential for osteoinduction, along with the inclusion of Sr and Zn; the release of ions also contributed to antimicrobial activity. Given the promising data and the need for bone graft materials for those with bone defects or at risk of fractures, this warrants further investigation. Further studies should include fine-tuning the degree of crosslinking of the hydrogel for improved osteogenesis and cell viability, as the degree of crosslinking appears to influence these outcomes. It would be further necessary to perform *in vivo* testing in conjunction with studies that fine-tune the hydrogel CL concentration to create a hydrogel with both favourable cell viability and calcium deposition for optimum bone regeneration outcomes, as tested using animal models.

## Conflicts of interest

The authors declare no conflicts of interest or competing financial interest.

## Data Availability

The raw original data are not publicly available due to an embargo period of 12 months. After this period, the data will be available upon reasonable request.
